# Disruption to water supply and waterborne communicable diseases in northeast Syria: a spatiotemporal analysis

**DOI:** 10.1186/s13031-023-00502-3

**Published:** 2023-02-04

**Authors:** Ruby Tabor, Naser Almhawish, Ibrahim Aladhan, Maia Tarnas, Richard Sullivan, Nabil Karah, Mark Zeitoun, Ruwan Ratnayake, Aula Abbara

**Affiliations:** 1 London School of Tropical Medicine and Hygiene, London, UK; 2 Assistance Coordination Unit, Gaziantep, Turkey; 3 Syrian Environmental Protection Agency, Gaziantep, Turkey; 4 University of California Irvine, Irvine, USA; 5 King’s College, London, UK; 6 Umea University, Umeå, Sweden; 7 University of East Anglia, Norwich, UK; 8 Geneva Water Hub, Geneva, Switzerland; 9 Department of Infectious Diseases, Imperial College, London, W2 1NY UK; 10 Syria Public Health Network, London, UK

## Abstract

**Background:**

In Syria, disruption to water and sanitation systems, together with poor access to vaccination, forced displacement and overcrowding contribute to increases in waterborne diseases (WBDs). The aim of this study is to perform a spatiotemporal analysis to investigate potential associations between interruptions to water, sanitation, and hygiene (WASH) and WBDs in northeast Syria using data collected by the Early Warning Alert and Response Network (EWARN) from Deir-ez-Zor, Raqqa, Hassakeh and parts of Aleppo governorates.

**Methods:**

We reviewed the literature databases of MEDLINE and Google Scholar and the updates of ReliefWeb to obtain information on acute disruptions and attacks against water infrastructure in northeast Syria between January 2015 and June 2021. The EWARN weekly trends of five syndromes representing waterborne diseases were plotted and analysed to identify time trends and the influence of these disruptions. To investigate a potential relationship, the Wilcoxon rank sum test was used to compare districts with and without disruptions. Time series analyses were carried out on major disruptions to analyse their effect on WBD incidence.

**Results:**

The literature review found several instances where water infrastructure was attacked or disrupted, suggesting that water has been deliberately targeted by both state and non-state actors in northeast Syria throughout the conflict. Over time, there was an overall upwards trend of other acute diarrhoea (OAD, *p* < 0.001), but downwards trends for acute jaundice syndrome, suspected typhoid fever and acute bloody diarrhoea. For the major disruption of the Alouk water plant, an interrupted time series analysis did not find a strong correlation between the disruption and changes in disease incidence in the weeks following the incident, but long-term increases in WBD were observed.

**Conclusions:**

While no strong immediate correlation could be established between disruptions to WASH and WBDs in northeast Syria, further research is essential to explore the impact of conflict-associated damage to civil infrastructure including WASH. This is vital though challenging given confounding factors which affect both WASH and WBDs in contexts like northeast Syria. As such, research which includes exploration of mitigation after damage to WASH is essential to improve understanding of impacts on quantity and quality of WASH. More granular research which explores the origin of cases of WBDs and how such communities are affected by challenges to WASH is needed. One step towards research on this, is the implementation of adequate reporting mechanisms for real time tracking of the WASH attacks, damages, direct effects, and likely impact in conjunction with environmental and public health bodies and surveillance systems.

## Introduction

Those living in areas of conflict are eight times more likely to lack basic drinking water services due to the breakdown of public services, damaged and neglected infrastructure, and the weaponization of water [1]. The use of water as a weapon to achieve military objectives, by controlling water systems or direct targeting of water infrastructure, date back over millennia [2]. In countries affected by protracted conflict where health systems are unable to meet the health or public health needs of the population, such as Syria, such attacks on water, sanitation, and hygiene (WASH) systems may lead to an increase in morbidity and mortality associated with waterborne infections. In northeast Syria, such attacks occur in already fragile WASH conditions where water availability has been degraded by climate-induced drought, reduction in flow of the Euphrates River and chronic reduction in groundwater levels with little slack in the system [3, 4].

Weaponization of water can be characterised in different ways relating to using water as a ‘military tool of domination and legitimacy’, or a ‘military target and goal’ to terrorise and enforce cooperation in civilians [5]. Zeitoun et al*.* have recently developed an analytical framework to facilitate the identification whether attacks are indiscriminate (not directed at a specific military objective) or discriminate (intentional and targeted at a specific military objective) and enable evaluation in terms of international norms and law [6]. This is more evident in contemporary conflicts where there is increased use of high yield munitions which penetrate the ground and have wider impacts on critical civil infrastructure, including for WASH as a secondary effect [7]. This has also occurred alongside the changing nature of warfare whereby there is a higher likelihood of ‘urban warfare’ with such heavy munitions used, leading to these secondary effects [8].

### Water and sanitation in Syria

Syria, like other countries in the Middle East and North Africa (MENA) region, is a water-scarce country, with a recorded average rainfall of less than 250 mm annually [9, 10]. Around 60% of Syria’s total water availability originates outside its borders, and five of its major rivers, including the Euphrates River, are shared with neighbouring countries, leading to a history of both cooperation and tension around the management of shared rivers [2]. The mismanagement of water for agriculture and lack of sustainable practices, especially between 1970 and 2000, were associated with declines in groundwater availability, leaving the country susceptible to drought [11]. Syria experiences hydrologic variability and has faced multiple droughts in recent history.

Before the conflict began, around 90% of Syria’s population had access to improved drinking water (GoS and UNO, 2010), referring to ways in which water is protected from the outside environment and available when needed; this either took the form of piped household water, public taps, tube wells, boreholes or dug wells or through identifying springs or mechanisms for rainwater collection (WHO and UNICEF, 2020). However, since the start of the conflict a lack of power supply, consumables, financial resources, and displacement of trained professionals has led to a significant deterioration of water infrastructure and access [2]. This has been exacerbated by direct and indirect attacks on water infrastructure in Syria [5, 12]. Currently, it is estimated that two thirds of Syria’s population do not have reliable access to safe drinking water. This has led to reliance on informal or private water providers such as water trucks, and has led to significant economic burdens on civilians, with Syrian households estimated to spend a quarter of their income on water [13].

A March 2022 briefing on the humanitarian effects of water shortages in northeast Syria noted significant, adverse impacts on livelihoods, electricity production, food security and health [4]. Reduction to water access has led to restrictions in hygiene practices e.g., bathing, and increased use of untreated water from wells, boreholes or water trucks increasing susceptibility to WBDs and skin diseases. Diarrhoeal illnesses together with increased food insecurity can also contribute to malnutrition which is increasingly seen in northeast Syria [4].

### Increased risk for waterborne disease and outbreaks

**Fig. 1:**
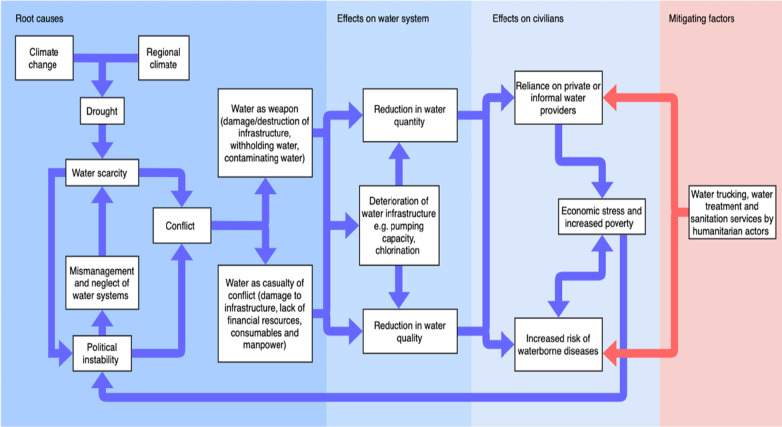
Framework showing how different factors, including climate change and conflict, lead to reductions in water quality and quantity

Children aged under 5 living in areas of protracted conflict are 20 times more likely to die from diarrhoeal diseases linked to unsafe water and sanitation than direct violence [1]. Multiple outbreaks of WBDs have been reported in Syria since the start of the conflict, often in displacement camps [14]. Despite outbreaks of WBDs there is very little published material on the associations between conflict and WBD incidence, particularly in northeast Syria. A recent paper by Abbara et al*.* [15] identified several attacks on water and water infrastructure in northwest Syria with an overall upwards trend of diarrhoeal diseases during this period. Attacks and disruptions such as these can have repeated impacts on the risk of waterborne disease outbreaks,we have summarised the potential pathways to impact in Fig. 1.

The aim of this study is to perform a spatiotemporal analysis to investigate potential associations between interruptions to WASH and WBDs in northeast Syria using data collected by the Early Warning Alert and Response Network (EWARN) from Deir-ez-Zor, Raqqa, Hassakeh and parts of Aleppo governorates..

## Methods

### Timeline of disruptive events

A literature review was conducted to identify events in which water infrastructure was disrupted or attacked in northeast Syria between January 2015 and June 2021. Examples of events include airstrikes on water pumping stations or towers and populations being physically cut off from water sources. Key words including “water”, “disruption”, “weaponization”, “waterborne disease”, “conflict”, “Syria'' and “northeast” were used to search MEDLINE and Google Scholar databases in English language for disruption events. For example, these included airstrikes impacting water stations [16]. Peer reviewed and grey literature including reports, flash updates and news articles were searched using the web portals of multilateral organisations such as the United Nations, World Health Organization, The United Nations Office for the Coordination of Humanitarian Affairs (UN-OCHA), Human Rights Watch, ReliefWeb, International Committee of the Red Cross (ICRC), Médecins Sans Frontières (MSF), United Nations Children’s Fund (UNICEF), and Save the Children. Information from grey literature was cross-checked across multiple courses to verify and triangulate reports. Details on the context and the duration of disruptions were also extracted where available, and a timeline of key events was constructed. Events were classified into “discriminate” when referring to purposeful attacks, or “indiscriminate” when occurring as a casualty of a wider attack. Verification calls with the Assistance Coordination Unit (ACU) team (including co-authors NA, IA) were also conducted to validate events reported in the literature. The events were recorded as a binary variable (0/1) in the weeks the disruption took place. Any event which did not specify a length of disruption was arbitrarily assumed to have a length of two weeks.

### Study population and period

**Fig. 2:**
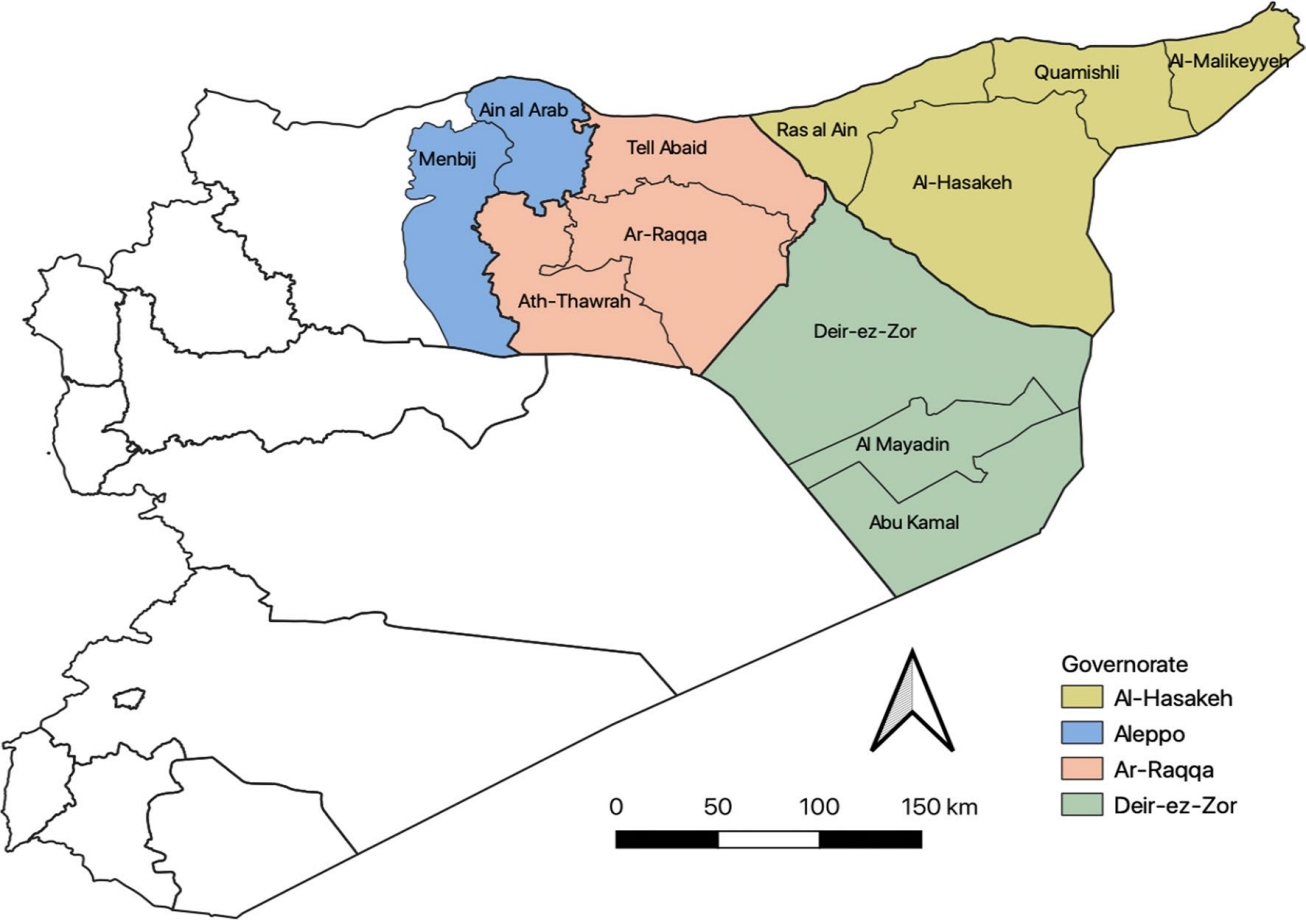
Map of Syria, highlighting the governorates and districts covered in this analysis

Our analysis covered the entire region of northeast Syria, including the governorates of Raqqa (Ar-Raqqa, Ath-Thawrah, Tell Abaid districts), Hassakeh (Al-Hasakeh, Al-Malikeyyeh, Quamishli, Ras al Ain districts), and Deir-ez-Zor (Deir-ez-Zor, Abu Kamal, Al Mayadin districts), and Aleppo (Ain al Arab, Menbij districts) (Fig. 2). We obtained data on WBD incidence covering the period between January 2015 (epiweek 1) to June 2021 (epiweek 24). Four districts (Ras al Ain, Quamishli, Al-Malikeyeh and Ain al Arab) did not have data reported until August 2015 (epiweek 34) due to a lack of coverage by EWARN up to this date.

### Population denominators

We computed district-level annual population estimates for northeast Syria in order to calculate disease incidence rates. Sources included the Humanitarian Needs Assessment Programme (HNAP) population estimates for < 15 year olds based on community focal point interviews, obtained from the ACU and extrapolated to whole populations based on an assumed population distribution of 45% under-15 s, and WorldPop annual estimates between 2015 and 2020, built on a predictive model that redistributes total census population into ~ 100 m^2^ grids based on remotely sensed population density (WorldPop, 2021). Estimates were weighted then averaged, using a quality score outlined in published guidance [17]. No population estimates were available for 2021, therefore estimates from 2020 were used.

### Disease data source

Syria currently has two parallel communicable disease surveillance systems, which are designed to improve rapid disease outbreak detection and control in emergency settings where routine surveillance is disrupted or non-existent [18]. Data on epidemic-prone diseases is collected through sentinel sites on weekly basis. EWARN is a non-governmental system established in 2013 by the Turkey-based ACU, a humanitarian non-governmental organisation, with support from the US Centres for Disease Control and Prevention and predominantly reports from areas outside of Syrian government control.

Data on weekly cases of WBDs were obtained from EWARN to a district level between January 2015 and June 2021. The WBDs were classified as: acute bloody diarrhoea (ABD; indicative of shigellosis), acute watery diarrhoea (AWD; cholera), acute jaundice syndrome (AJS; hepatitis A and E), suspected typhoid fever (STF), and any other acute diarrhoea (OAD). Total counts were collated by sex and age group (< 5 and ≥ 5). The syndromic case definitions and alert thresholds are outlined in Appendix 1. Weekly incidence by district was calculated as total disease counts divided by the population denominator estimates and multiplied by 100,000.

### Seasonal decomposition analysis

In order to better understand and compare the trends and seasonality for each disease overall and between districts, time series were separated into seasonal components, using the STL function in R [19]. An additive time series model was used, where temporal changes remain relative. After estimating the trend and seasonal pattern, the model was checked by calculating the residual series which, if the model fully accounts for the other components, should be stationary.

### Relationship between annual incidence and presence of disruptions

Districts were categorised as those where there had been disruptions during a certain period, during the entire period, or no disruptions reported. The Wilcoxon rank sum test with continuity correction was conducted to compare trends in all WBDs between districts with and without disruptions.

### Interrupted time series analysis

Where a major disruption was identified in a given district(s), an interrupted time series analysis was carried out to investigate the effects of disruptions on WBD incidence by comparing weekly incidence in affected districts one year before and after the disruption in order to explore the effects of long-scale interruptions to water infrastructure [20]. We focused on the longest and most widespread disruption to Alouk water station which affected water availability in Al-Hasakeh district from October 2019 onwards [21]. As seasonality was not found to have a fixed parametric form which could be fully modelled (i.e., the linear model had a poor fit), a natural cubic spline method was used.

This model was carried out for the period both before and after the disruption, with a regression line drawn to show the fitted values and overall trend. A lag or transition period of 3 weeks was introduced to between the “before” and “after” periods to account for the biological and behavioural aspects of infection—for example, to account for multiple incubation periods (i.e. generations) for each WBD, and/or the lag in adopting new water-seeking behaviours in the face of the disruption.

## Results

### Attacks and disruptions to water infrastructure

**Fig. 3:**
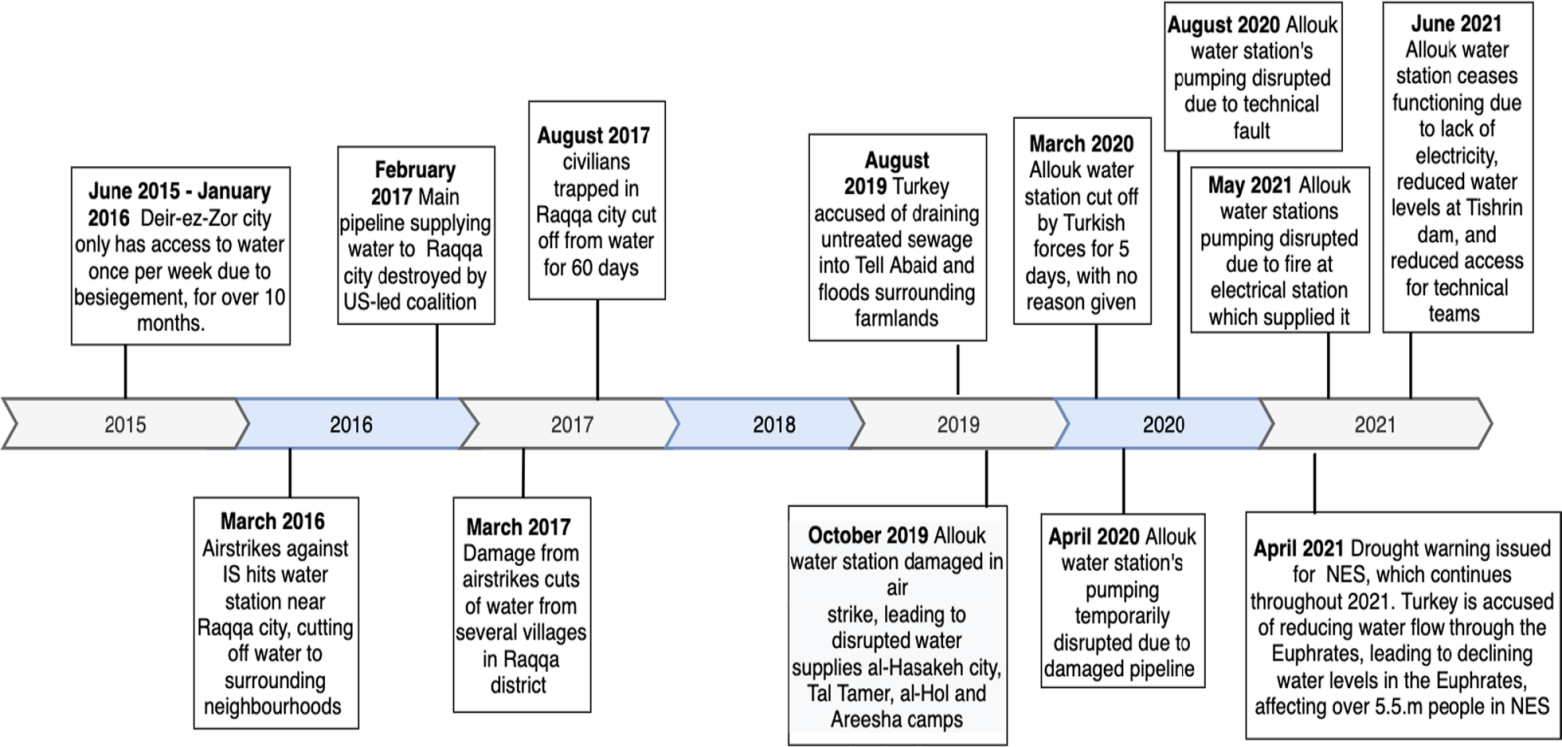
Timeline of disruption events between 2015 and 2021 in northeast Syria with details available. Data were triangulated from different sources to cross-check disruption events

14 large-scale individual attacks and disruptions against water infrastructure were identified between January 2015 and June 2021. These are displayed in a timeline of events in Fig. 3 and Appendix 1. The disruption events appeared to be clustered in space and time, with reported instances occurring in Deir-ez-Zor city from 2015 to 2016, Raqqa governorate from 2016 to 2017, and Hassakeh governorate from 2019 to June 2021. Both Deir-ez-Zor and Ar-Raqqa cities experienced severe water shortages for the civilians trapped inside the cities during besiegement from opposing forces. Deir-ez-Zor’s water shortage was likely a casualty of the destruction of the city’s infrastructure, as there was insufficient electricity for water pumping. However, given the targeted attack on its main pipeline by coalition forces, Ar-Raqqa’s water shortage was more likely to be a case of purposeful interference.

### Waterborne diseases in northeast Syria

#### Descriptive analysis of waterborne diseases in northeast Syria

**Fig. 4:**
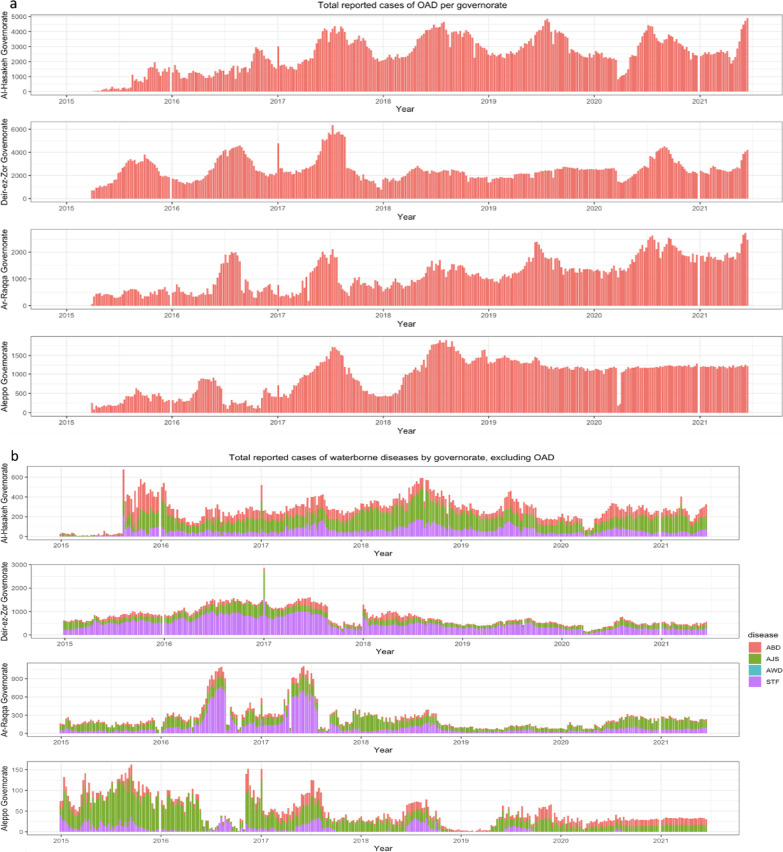
Total reported cases of OAD—(other acute diarrhoea) (**a**) and ABD (acute bloody diarrhoea), AJS (acute jaundice syndrome), AWD (acute watery diarrhoea) and STF (suspected typhoid fever) (**b**) as reported weekly, by governorate

A total of 2,761,015 suspected cases of waterborne disease were reported in northeast Syria between January 2015 and June 2021. Of these, OAD accounted for the majority (84%) of reported cases (Table 1). Overall, 56% of all suspected cases were reported in children under 5, making up most cases of ABD, OAD and AJS cases. In contrast, the majority of ABD and STF cases were detected in those aged over 5. Only 8 cases of AWD, representing suspected cholera, were identified, all of which were in Ar-Raqqa district between July 2017 and January 2018. Across the entire study period, districts reported markedly different trends in WBDs (Fig. 4). Al Mayadin district had the highest median weekly incidence of WBD (305.2 (IQR 207.5, 364.0) per 100,000), while Ain al Arab had the lowest (41.5 (IQR 19.3, 74.5) per 100,000). Males accounted for 51% of the total suspected cases, and there was no significant difference by sex for any WBD other than AWD (due to the small sample size, this may be due to chance).

**Table 1:** number of reported cases of each waterborne disease, stratified by age and sex

Characteristic		AWD	ABD	OAD	AJS	STF
Age	< 5	1 (13%)	46,444 (61%)	1,374,602 (60%)	88,792 (56%)	36,497 (17%)
	≥ 5	7 (88%)	29,316 (39%)	932,011 (40%)	69,076 (44%)	184,279 (83%)
Sex	Male	3 (38%)	39,942 (53%)	1,175,360 (51%)	82,362 (52%)	108,316 (49%)
	Female	5 (63%)	35,818 (47%)	1,131,253 (49%)	75,506 (48%)	112,460 (51%)
Total		8	75,750	2,306,613	157,868	220,776

*AWD* acute watery diarrhoea, *ABD* acute bloody diarrhoea, *OAD* other acute diarrhoea, *AJS* acute jaundice syndrome, *STF* suspected typhoid fever

#### Population denominators

From our estimates of annual population, there was considerable variation between years and districts, illustrating dynamic population changes throughout the study period, potentially due to displacement. For example, large drops in population were seen in Deir-ez-Zor, Ar-Raqqa and Al-Mayadin districts between 2017 and 2018, with population decreasing between years by 33%, 37% and 41.5% respectively. However, while the total population saw a decrease of 13%, other districts such as Tell Abaid and Al-Hasakeh saw increases in by as much as 39% between 2017 and 2018, illustrating that these changes did not occur across the entire region, and indicating possible population movement between districts.

#### Other acute diarrhoea

**Fig. 5:**
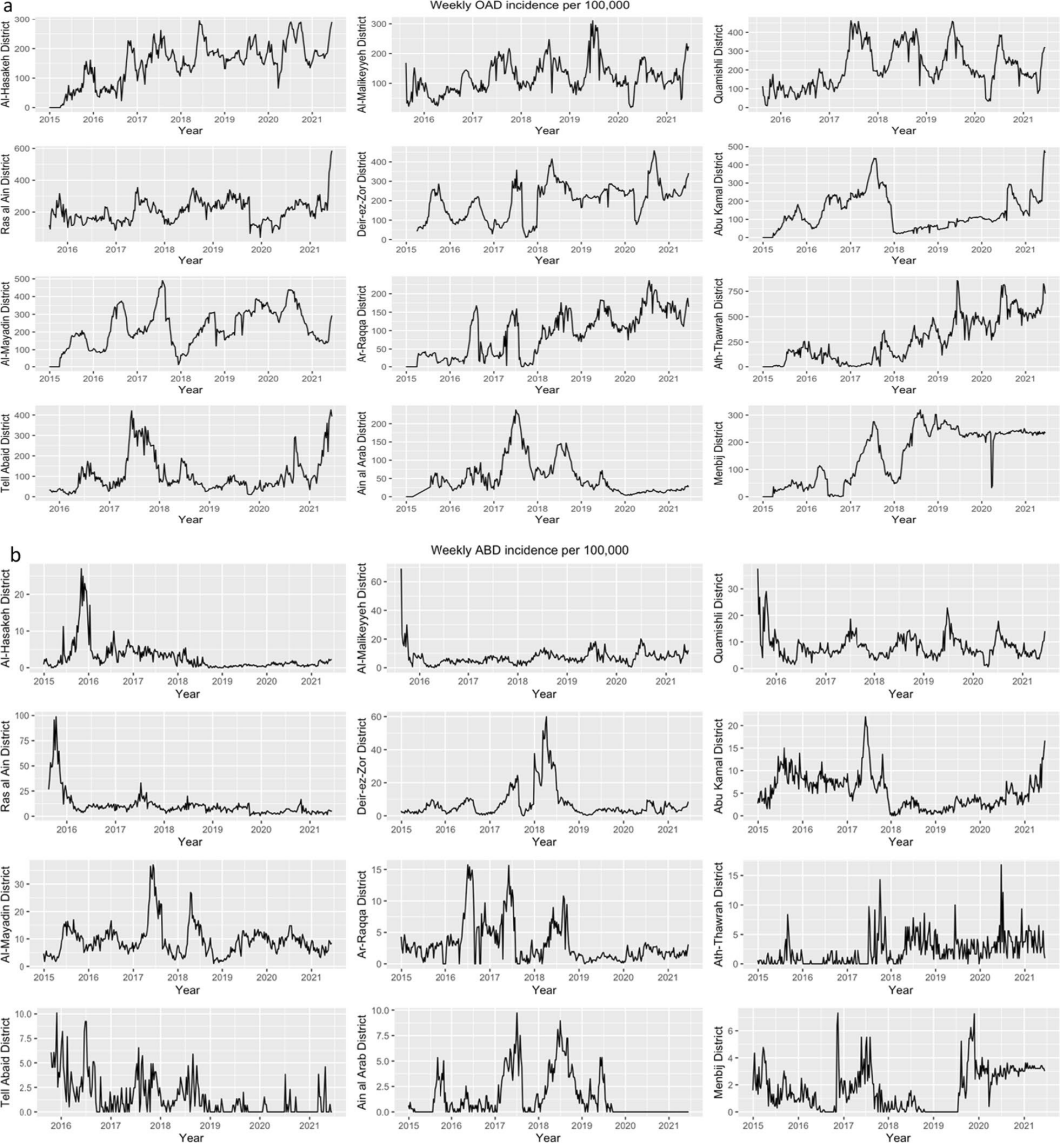

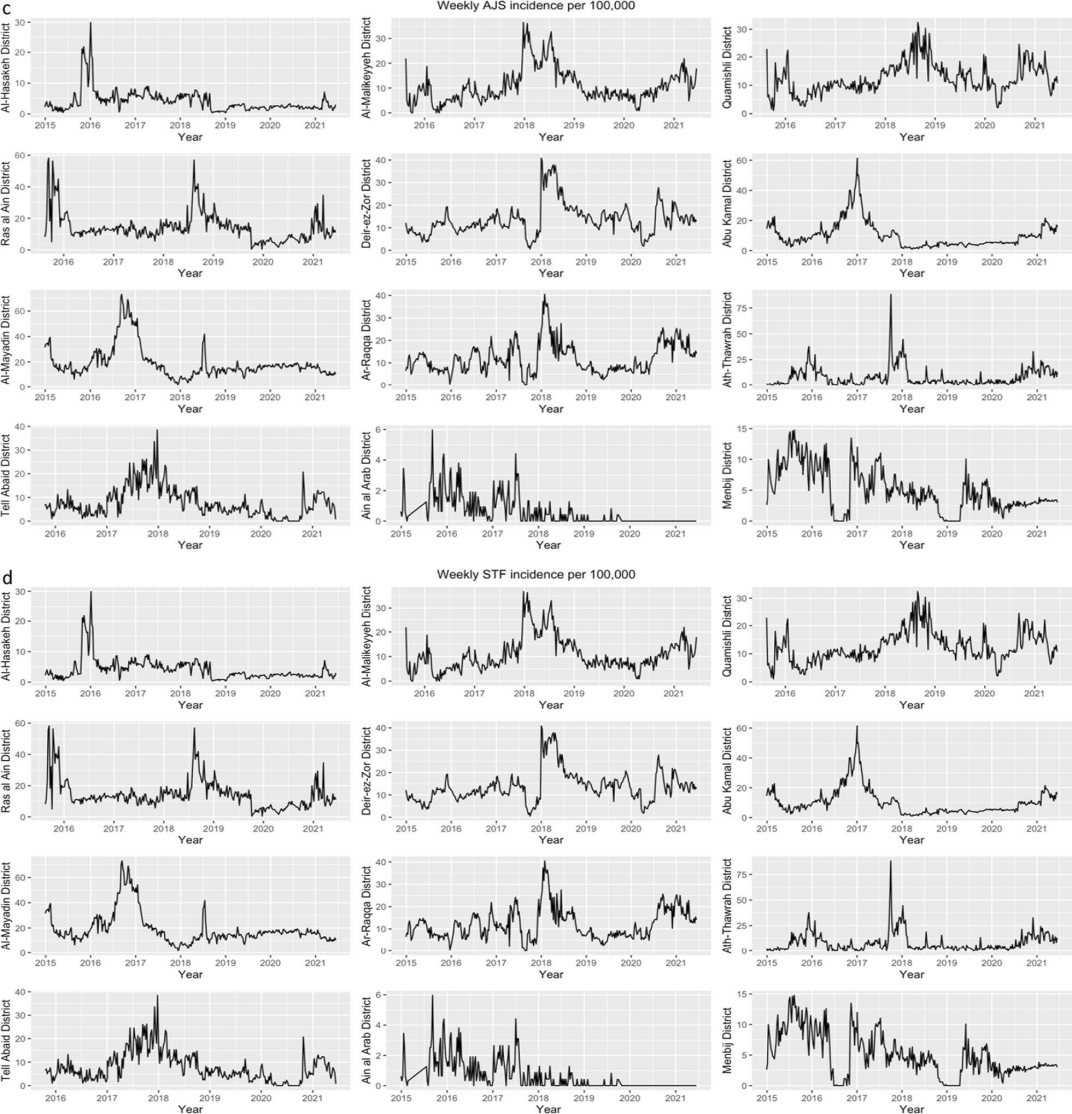
Weekly incidence between 2015 and 2021, per district of OAD (other acute diarrhoea) (**a**); ABD (acute bloody diarrhoea) (**b**); AJS (acute jaundice syndrome) (**c**); STF (suspected typhoid fever) (**d**)

After adjusting for seasonality, a positive upwards trend of OAD over time was identified in all districts other than Ain al Arab, for which a negative downwards trend was observed. Abu Kamal saw a significant decrease in OAD incidence around the beginning of 2018, which could not be explained by any drop in population. Ath-Thawrah had the highest median weekly incidence across the entire period (233.6 (IQR 93.2, 454.9) per 100,000), while the lowest was observed in Ain al Arab (39.4 (IQR 18.9, 55.2) per 100,000). See Fig. 5a.

#### Acute bloody diarrhoea

When adjusted for seasonality, upward trends were found in Al-Malikeyyeh, Abu Kamal, Al Mayadin, Ath-Thawrah, and Menbij districts. Deir-ez-Zor saw a large peak in cases throughout 2018. Districts in Hassakeh governorate experienced a large peak in late August, after which weekly incidence fell to a baseline of under 20 cases per 100,000. During this year, there was also a large drop in population in the district, falling from 830,606 to 557,363 according to our estimates, which also coincided with conflict within the district and resulting population movement. See Fig. 5b.

#### Acute jaundice syndrome and suspected typhoid fever

AJS and STF saw a large amount of variation across different districts. Little seasonality was observed in AJS incidence, however peaks in either 2017 or 2018 were observed in the majority of districts, and from mid-2020 onwards there appeared to be the beginning of an upwards trend in almost all districts other than Al-Mayadin and Ain al Arab. Strong seasonal patterns in STF were seen in a number of districts, in particular Al-Hasakeh. In Abu Kamal and Al-Mayadin, large drops in incidence were observed between mid-2017 and early 2018. Changes in population could not be attributed to this pattern: this was a gradual decrease rather than a sudden drop, and population denominators decreased between 2017 and 2018, which would be expected to lead to an increase, rather than decrease in incidence. Comparatively, a large increase in 2018 was observed in Deir-ez-Zor, which given its population decrease between 2017 and 2018, is very likely due to population changes. See Fig. 5c, d.

### Waterborne diseases and disruptions to water infrastructure in northeast Syria

To investigate the potential link between WBD incidence and disruptions to water infrastructure, we focused on districts in which disruption events had been found in the literature: Al-Hasakeh, Deir-ez-Zor, Ar-Raqqa, and Tell Abaid. The difference in median incidence of districts with and without disruptions showed that districts which had not experienced disruptions were mixed. Generally, a significantly higher median WBD incidence was found in districts without interruptions compared to those with disruptions (Table 2). However, a major unmeasured confounder was poorer reporting capacity for EWARN in areas under attack. All diseases other than ABD had a significantly higher median incidence in districts which had experienced disruptions in 2015, suggesting that events which affected Deir-ez-Zor in 2015 may have had a significant effect on WBD incidence. AJS and STF also had a significantly higher median incidence in districts which had experienced disruptions in 2016 and 2021.

**Table 2:** Median weekly incidence per 100,00 per disease per year between districts which had experienced disruptions to water infrastructure and districts which had not

Disease	Year	Median (IQR) weekly disease incidence (per 100,000)		*P* value	Significantly higher
		Disruption	No disruption		
ABD	2015	3.18 (2.18–5.87)	3.02 (1.01–8.41)	0.4225	–
	2016	4.70 (3.27–7.97)	3.77 (0.95–7.42)	< 0.05	Disruption
	2017	3.63 (0.82–7.61)	4.87 (2.23–8.16)	0.07907	–
	2019	0.37 (9.00–0.59)	3.24 (1.04–6.49)	< 0.001	No disruption
	2020	0.89 (0.71–1.25)	3.10 (1.27–6.01)	< 0.001	No disruption
	2021	1.69 (0.83–1.83)	4.72 (3.22–6.67)	0.00266	No disruption
	Total	2.42 (0.71–5.21)	7.02 (4.72–8.61)	< 0.001	No disruption
OAD	2015	104.64 (31.81–201.31)	37.90 (0.15–102.06)	< 0.001	Disruption
	2016	31.57 (20.95–59.06)	89.85 (53.01–148.32)	< 0.001	No disruption
	2017	31.99 (8.64–109.96)	170.47 (95.61–247.45)	< 0.001	No disruption
	2019	118.88 (52.64–169.25)	216.13 (109.65–271.96)	< 0.001	No disruption
	2020	206.34 (152.10–251.58)	182.15 (102.95–253.75)	0.1682	–
	2021	181.94 (157.08–239.92)	201.07 (136.26–243.57)	0.08889	–
	Total	129.75 (53.65–226.59)	162.40 (110.20–231.50)	< 0.001	No disruption
AJS	2015	9.19 (6.98–12.16)	7.41 (3.01–12.58)	< 0.05	Disruption
	2016	11.44 (7.56–14.61)	8.21 (3.81–12.31)	< 0.001	Disruption
	2017	10.29 (5.15–16.36)	9.42 (5.40–13.93)	0.4894	–
	2019	2.90 (2.01–4.68)	6.86 (3.34–12.99)	< 0.001	No disruption
	2020	2.32 (2.14–2.67)	6.30 (2.62–12.16)	< 0.001	No disruption
	2021	12.29 (4.61–14.81)	11.22 (4.57–15.63)	< 0.05	Disruption
	Total	6.53 (2.65–13.03)	11.14 (7.59–15.30)	< 0.001	No disruption
STF	2015	23.69 (15.88–40.06)	3.59 (0.88–8.62)	< 0.001	Disruption
	2016	11.04 (7.97–19.41)	3.53 (0.88–17.34)	< 0.001	Disruption
	2017	12.45 (2.97–43.07)	5.77 (2.2–20.57)	0.06546	–
	2019	2.53 (1.33–4.01)	4.64 (1.63–6.74)	< 0.001	No disruption
	2020	1.25 (0.89–1.65)	5.43 (1.05–9.35)	< 0.001	No disruption
	2021	5.96 (2.26–14.68)	4.34 (0.00–8.402)	< 0.001	Disruption
	Total	4.95 (1.14–15.72)	4.30 (1.96–7.20)	< 0.001	Disruption
All WBDs	2015	128.26 (58.81–265.44)	54.64 (25.49–139.92)	< 0.001	Disruption
	2016	59.77 (44.75–103.40)	113.79 (64.03–184.79)	< 0.001	No disruption
	2017	60.61 (21.67–170.46)	192.10 (119.6–317.00)	< 0.001	No disruption
	2019	124.46 (61.50–175.33)	231.88 (120.91–318.58)	< 0.001	No disruption
	2020	210.26 (157.36–256.88)	206.94 (115.93–284.86)	0.7574	–
	2021	187.20 (182.20–197.40)	226.08 (161.08–284.19)	0.1921	–
	Total	196.16 (175.16–223.90)	229.60 (159.39–272.61)	0.1001	–

**Fig. 6:**
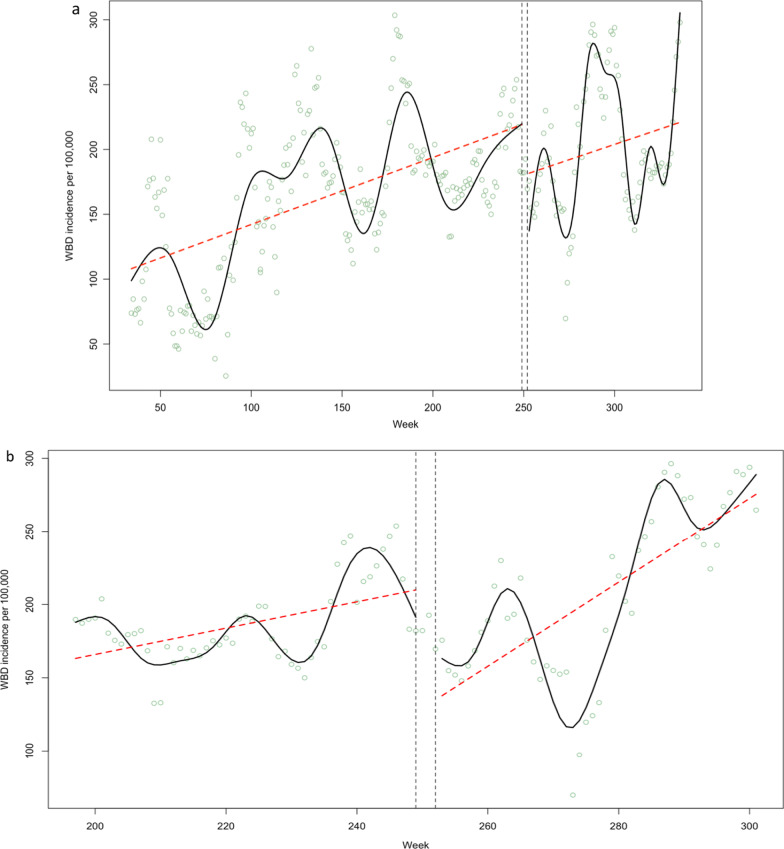
Weekly incidence of WBDs (waterborne diseases) in Al-Hasakeh before and after the disruption (denoted by vertical dotted lines). Green dots indicate weekly incidence, red lines indicate the linear regression line, and black lines indicate the fitted cubic spline values. (**a**) The entire reporting period and (**b**) a year before and after the disruption (weeks 197–301)

An interrupted time-series analysis was carried out in order to visualise trends in data before and after major disruptions. Trends in WBDs incidence in Al-Hasakeh before and after the start of Operation Peace Spring and the capture of Alouk water station are visualised in Fig. 6, with trends over the whole period and during the year before and after the main event plotted. Immediately following the disruption, there appears to be an overall lower incidence for the rest of the reporting period, reflecting the findings of the Wilcoxon rank sum test. This may indicate a true decrease in incidence or alternatively, the lower capacity of EWARN to detect WBD after the attack. When examining the context of the year before and after the start of the disruption, a steeper increase in level and slope was observed, with higher overall incidence observed during the year after the disruption. This suggests that there may have been a more short-term relationship between the disruption and WBD incidence and/or reporting, with cases initially dropping (or being underreported), before going back to baseline levels and increasing. This may represent a long-term impact of the water station disruption that is not immediately discernible in the weeks following the attack. Alternatively, when looked at in the context of the entire reporting period, there appears to be a level change, with overall WBD incidence dropping to a baseline below that of the incidence before the disruption, potentially indicating the influence of seasonality and random variation in WBD trends.

## Discussion

Purposeful destruction of civilian water infrastructure is a violation of international law [22, 23]. However, water interruption during armed conflict may also occur indirectly as a result of the use of heavy weapons which can cause indirect but significant damage to essential infrastructure. Such ‘explosive weapons with wide area effects’ as termed by the International Committee of the Red Cross (ICRC) have a myriad of effects on civilians and disruption to services including electricity, WASH and healthcare [24]. The long-term health impacts of such damage are harder to quantify but the use of heavy munitions is increasing in modern armed conflicts.

From our literature review, we noted 14 disruption events in Ar-Raqqa, Deir-ez-Zor and Al-Hasakeh cities, which were the most populous cities in the region and have been the focal point of the conflict to date. Similar events and findings were reported by Abbara et al. [15] in their investigation into the weaponization of water in northwest Syria in Aleppo and Idlib governorates, with the majority of events occurring in the main cities.

Interference with water also occurs elsewhere in the MENA region such as in Palestine where pumping rates and water consumption differ significantly between Israel and Palestine despite the two nations despite sharing aquifers, with evidence noted that Israel regularly violated the limits set out in the Oslo Agreement and that the countries geographical advantage in access to water allowed it to exploit Palestine [25]. As a result, a framework of “hydro-hegemony” was conceptualised, which addresses the role that power asymmetry plays in creating and maintaining transnational conflict over shared water sources [26]. Spiegel et al.’s investigation into cholera in Yemen also noted the impacts of insecurity (specifically, airstrikes) and conflict on preparedness planning and community-directed responses [27]. For northeast Syria, interference with WASH will have an increasingly important influence due to water sources and increasing water shortages.

### Disease and surveillance data

That 80% of disease reports were “other acute diarrhoea” deserves some attention to ensure accurate reporting of the case definitions for acute bloody and acute watery diarrhoea by health workers. An overall upwards trend of WBDs, with seasonal peaks in the late summer, was observed throughout the whole region, corresponding to trends reported in northwest Syria [15]. However, considerable variability in trends and median weekly levels of WBDs were observed in different districts. Trends of waterborne diseases in areas of conflict are likely to be affected by various factors such as conflict, reporting accuracy and population movement [13]. Establishing cause and effect is geo-temporally complex. For example, Ain al Arab district consistently reported some of the lowest median incidence of WBDs, despite having relatively high completeness and timeliness throughout the study period (data not published), indicating potential differences in detection and reporting capacity. A previous study using survey data on WASH access in southern Syria found that a significant risk factor for the incidence of childhood diarrhoea was whether the household shelter of the responder was damaged. Significant protective factors were found to be hygiene access and reported handwashing within the household [28]. Therefore, it is likely that factors including hygiene access and living conditions may be important factors in the incidence of WBD.

The risk of cholera is also greatly increased in areas of conflict; in Yemen, targeting of hospitals, clinics and water infrastructure led to the largest cholera outbreak in the world [27]. Despite concerns about the potential for cholera to spread in Syria due to the poor water and sanitation levels, outbreaks in neighbouring Iraq, and unreliable disease surveillance [29], AWD (indicative of cholera) had very low case levels in northeast Syria. There was one alert of an RDT-positive case in 2015 by EWARN for which no culture could be obtained due to ongoing conflict. Here, the lack of outbreak detection may be explained by a failure to initiate regular investigation and response protocols due to lack of access [30]. However, in our analysis, only 8 cases were reported across the entire study period and in two alert-level events in Raqqa. This may represent sporadic cases without further transmission or a failure to initiate regular investigation and response due to limited access or resources [30].

### Relationship between disruptions to water infrastructure and waterborne diseases

The relationship between weaponization of water and waterborne diseases has been investigated in a small number of reports. In northwest Syria, the peak of disease incidence occurred during an intensification of attacks on water infrastructure, although no direct correlation could be drawn [15]. A broader relationship between conflict, quantified by records of improvised explosive devices, and incidence of polio in Afghanistan has also been reported. The association was thought to be predominantly due to disruption to childhood polio vaccination due to the conflict. However, causation could again not be established due to data constraints [31].

Our hypothesis is that disruptions to water infrastructure could lead to increased incidence of WBDs, perhaps extending to months or a year after the disruption. This appears to be a possibility for Alouk water station which had repeated and prolonged interruptions. For the other 13 disruption events noted in the literature, there was no clear evidence of short- or longer-term effects and therefore we did not attempt an interrupted time series analysis. This may be for a number of reasons including insufficient information in the literature about the extent and duration of disruptions to water during the study period which speaks to the need for systematic and more accurate conflict mapping; that investigated WBDs to district level gives insufficient granularity thereby missing effects at the subdistrict level; decreased detection and reporting of the EWARN system particularly during escalations of conflict; the use of mitigating factors e.g. water trucking which averted WBDs outbreaks; the difficult in isolating the population impacted for a water system which may extend to several sub-districts. It may also be due to mitigating factors including the supplementation of piped water supply by vendors; in such instances, affordability rather than availability may become an important factor. Additionally, it may be that the repeatedly affected population may already be chronically water insecure such that the impact of further, acute disruption could not be elicited.

### Limitations

An important limitation in this study is the lack of credible, consistent reports of attacks against WASH. Unlike the case for Yemen, where the Yemen Data Project (https://yemendataproject.org/) systematically compiles verified lists of air raids, no similar efforts are in place in Syria [27]. Despite triangulation to verify sources where possible, the majority of sources used were grey literature or news articles, few published accounts; without a systematic process, we suspect a high degree of undercapture of events. For WBDs, there may have been under-reporting into EWARN, especially during periods of violent attacks. Our data analysis went to the district level, potentially missing changes at the subdistrict level. It is also known that many in northeast Syria, particularly in camp settings, rely on trucked water from either private companies or by humanitarian actors [32]. Additionally, water supplies may be provided by vendors or from private wells, in such situations affordability rather than availability becomes the limiting factor for the population. Such factors may therefore mitigate the effects of interruptions to WASH in the area and are harder to account for. The WASH cluster collates some information on water supply and water quality at point of use. Lastly, our population denominators are estimates given the lack of one regional civil record with accurate numbers in this context.

2022 has seen a significant increase in cases of cholera including in countries affected by armed conflict and humanitarian crises [33], this includes in Syria where a cholera outbreak was declared on 10th September 2022, with most cases occurring in the northeast [34]. This emphasises the importance of research which explores conflict, WASH and WBDs.

## Conclusion

Despite limitations, interruptions to WASH in northeast Syria and changes to WBDs are noted over the time period of study. It is however difficult to establish a causality relationship given multiple factors which likely have an impact on WASH in the region. However, conflict and communicable diseases [35], particularly WBDs [36–38], do have an established association given the extensive damage which increasingly occurs to WASH infrastructure, particularly in modern conflicts. This is noted in the increase in cholera outbreaks noted in 2022, including in a number of conflict and post-conflict countries. We suggest that similar reporting mechanisms to WHO on healthcare can be implemented for attacks on civic infrastructures during conflict, particularly WASH given the health impacts, including the potential to result in epidemics, of such attacks. Though this report could not draw a direct link between attacks and increases in WBDs incidence, it acts as a starting point for more comprehensive linking of the health impacts of conflict with downstream health data. Such evidence can be utilised to challenge international norms and military doctrine, outside the overt violations of IHL which occur during conflict.

## Data Availability

The datasets used and/or analysed during the current study are available from the corresponding author on reasonable request.

## Appendix 1

See Table

**Table 3:** Diseases and syndromes of waterborne diseases reported by EWARN sentinel sites. [39] Adapted from EWARN (Early Warning and Response Network) guidelines

Disease/syndrome	Code	Syndromic case definitions	Alert threshold
Other acute diarrhoea	OAD	Any person with acute diarrhoea (three or more loose stools in the past 24 h), not due to bloody diarrhoeal or suspected cholera	Double the average of the last 3 weeks in a given location
Acute bloody diarrhoea (suspected shigellosis)	ABD	Acute diarrhoea (three or more abnormally loose or fluid stools in the past 24 h) with visible blood in stool (preferably observed by the clinician)	≥ 5 cases in 1 location in 1 week
Acute watery diarrhoea (suspected cholera)	AWD	Any person aged 5 years or more with severe dehydration OR death from acute watery diarrhoea in the past 24 h, with or without vomiting	One case
Acute jaundice syndrome (hepatitis A and E)	AJS	Acute onset of jaundice (yellowing of sclera of eyes or skin or dark urine), AND severe illness (fatigue, nausea, vomiting and abdominal pain) AND the absence of any known precipitating factors	≥ 5 cases in 1 location in 1 week
Suspected typhoid fever	STF	Any person with acute illness and fever of at least 38 °C For 3 or more days with abdominal symptoms (diarrhoea or constipation or abdominal tenderness progressing to prostration) and relative bradycardia OR symptomatic case contacted with confirmed case	≥ 5 cases in 1 location in 1 week

3.

## Abbreviations

ABD: Acute blood diarrhoea
AJS: Acute jaundice syndrome
EWARN: Early warning alert and response network
OAD: Other acute diarrhoea
STF: Suspected typhoid fever
WASH: Water, sanitation and hygiene
WBDs: Waterborne diseases
WHO: World Health Organisation
